# Pamiparib in patients with locally advanced or metastatic HER2-negative breast cancer with germline *BRCA* mutations: a phase II study

**DOI:** 10.1007/s10549-022-06785-z

**Published:** 2022-12-02

**Authors:** Binghe Xu, Tao Sun, Yanxia Shi, Jiuwei Cui, Yongmei Yin, Quchang Ouyang, Qiang Liu, Qingyuan Zhang, Yiding Chen, Shouman Wang, Xiaojia Wang, Zhongsheng Tong, Yahua Zhong, Jiayu Wang, Min Yan, Xi Yan, Chuan Wang, Jifeng Feng, Xiuli Wang, Gang Hu, Ying Cheng, Ruimin Ge, Zhaoyin Zhu, Wa Zhang, Zhimin Shao

**Affiliations:** 1 National Cancer Center, Cancer Hospital, Chinese Academy of Medical Sciences and Peking Union Medical College, Beijing, China; 2 Department of Breast Medicine, Cancer Hospital of China Medical University, Cancer Hospital of Dalian University of Technology, Liaoning Cancer Hospital and Institute, Shenyang, China; 3 Department of Medical Oncology, Sun Yat-Sen University Cancer Center, The State Key Laboratory of Oncology in South China, Collaborative Innovation Center for Cancer Medicine, Guangzhou, China; 4 Cancer Center, The First Hospital of Jilin University, Changchun, China; 5 Department of Oncology, Jiangsu Province Hospital, Nanjing, China; 6 Department of Breast Medicine, Hunan Cancer Hospital, Changsha, China; 7 Breast Tumor Center, Sun Yat-Sen Memorial Hospital, Sun Yat-Sen University, Guangzhou, China; 8 Department of Medical Oncology, Harbin Medical University Cancer Hospital, Harbin, China; 9 Department of Surgical Oncology, The Second Affiliated Hospital, Zhejiang University School of Medicine, Hangzhou, China; 10 Department of Breast Surgery, Xiangya Hospital, Central South University, Changsha, China; 11 Department of Medical Oncology, Zhejiang Cancer Hospital, Hangzhou, China; 12 Department of Breast Oncology, Tianjin Medical University Cancer Institute and Hospital, Tianjin, China; 13 Zhongnan Hospital of Wuhan University, Wuhan, China; 14 Department of Breast Disease, Henan Breast Cancer Center, The Affiliated Cancer Hospital of Zhengzhou University and Henan Cancer Hospital, Zhengzhou, China; 15 West China Hospital of Sichuan University, Chengdu, China; 16 Department of Breast Surgery, Fujian Medical University Union Hospital, Fuzhou, China; 17 Department of Medical Oncology, Jiangsu Cancer Hospital, Nanjing, Jiangsu China; 18 The Second Hospital of Jilin University, Jilin, China; 19 Department of Breast Surgery, Sichuan Provincial People’s Hospital, University of Electronic Science and Technology of China, Chengdu, Sichuan China; 20 Department of Medical Thoracic Oncology, Jilin Provincial Cancer Hospital, Changchun, China; 21 Clinical Development, BeiGene Ltd, Beijing, China; 22 BeiGene USA Inc, Ridgefield Park, NJ USA; 23 Department of Breast Surgery, Fudan University Shanghai Cancer Center, Shanghai, China

**Keywords:** Clinical trial, Breast cancer, PARP inhibitor, Pamiparib, *BRCA* mutation

## Abstract

**Purpose:**

To evaluate the efficacy and safety of pamiparib in patients with locally advanced or metastatic human epidermal growth factor receptor 2-negative (HER2−) breast cancer, with deleterious or suspected deleterious germline *BRCA1/2* mutations (g*BRCA1/2* m).

**Methods:**

In this open-label, phase II, multicenter study in China (NCT03575065), patients with triple-negative breast cancer (TNBC cohort) or hormone receptor-positive (HR+)/HER2− breast cancer (HR+/HER2− cohort) and ≤ 2 prior lines of chemotherapy received pamiparib 60 mg orally twice daily in 28-day, continuous cycles. The primary endpoint was objective response rate (ORR; RECIST v1.1) by independent review committee.

**Results:**

In total, 88 patients were enrolled (TNBC cohort: 62; HR+/HER2− cohort: 26). Median age was 45.5 (range: 27–67) years, and 60 patients (68.2%) had received 1 or 2 prior lines of chemotherapy; 42 patients (47.7%) had previously received platinum chemotherapy. In the TNBC cohort, ORR was 38.2% (95% confidence interval [CI] 25.4–52.3) and median duration of response (DoR) was 7.0 months (95% CI 3.9–not estimable). In the HR+/HER2− cohort, ORR was 61.9% (95% CI 38.4–81.9) and median DoR was 7.5 months (95% CI 5.6–14.8). The most common treatment-emergent adverse events (TEAEs), treatment-related TEAEs, and ≥ Grade 3 TEAEs were hematologic (including anemia, decreased neutrophil count, and decreased white blood cell count). Overall, 64.8% of patients had TEAEs leading to dose reduction and 2.3% had TEAEs leading to treatment discontinuation.

**Conclusion:**

Pamiparib showed encouraging efficacy and an acceptable safety profile in patients with locally advanced and metastatic HER2− breast cancer with g*BRCA1/2* m.

**Trial registration:**

ClinicalTrials.gov, NCT03575065; July 2, 2018.

**Supplementary Information:**

The online version contains supplementary material available at 10.1007/s10549-022-06785-z.

## Introduction

In 2020, breast cancer was the most frequently diagnosed cancer worldwide, and was the leading cause of cancer mortality in women [1]. Triple-negative breast cancer (TNBC) is characterized by the absence of estrogen and progesterone receptors, and human epidermal growth factor receptor 2 (HER2) expression, and accounts for 15–20% of breast cancer cases [2]. Hormone receptor-positive/HER2-negative (HR+/HER2−) tumors represent 65–70% of all breast cancers [3], with 9–10% of patients harboring germline breast cancer susceptibility gene (*BRCA)* mutations [4, 5]. Treatment for TNBC with first-line chemotherapy provides only limited therapeutic benefit, while reported objective response rates (ORRs) after receiving second- or later-lines of chemotherapy are varied, but typically poor (e.g., 6–18%) [6]. For HR+/HER2− breast cancer, endocrine therapy is normally recommended for advanced disease, with or without targeted therapy (cyclin-dependent kinase 4/6 inhibitors or everolimus) [7]. However, some patients ultimately develop resistance after several lines of endocrine treatment [7]. Thus, there is a need for more efficacious treatment options in advanced HER2− breast cancer.

Studies investigating poly (ADP-ribose) polymerase (PARP) inhibitors (PARPi) have demonstrated prolonged progression-free survival (PFS) and improved ORR versus chemotherapy in patients with metastatic HER2− breast cancer with germline *BRCA* mutations who had received prior chemotherapy in the neoadjuvant, adjuvant, locally advanced, or metastatic settings [8, 9]. These studies led to the approval of olaparib and talazoparib by the US Food and Drug Administration and European Medicines Agency for patients with locally advanced or metastatic HER2− breast cancer with germline *BRCA* mutations [10–13]. The advent of PARPi represented the first availability of targeted treatments for patients in this setting [14]. In China, however, despite approvals for PARPi for the treatment of ovarian cancers [15–17], there are no approved PARPi for the treatment of advanced HER2− breast cancer with germline *BRCA* mutations. There is currently only one other study reporting the efficacy and safety of PARPi in this breast cancer patient population in China [18].

Pamiparib is a novel, investigational, small molecule inhibitor of PARP1/2 that has demonstrated potent antitumor activity, brain penetration, and PARP-DNA complex trapping in preclinical models [19]. Unlike other PARPi, pamiparib is not a substrate of P-glycoprotein, and so delivery across the blood–brain barrier may be less restricted [19], which is currently under investigation in a clinical trial (NCT04614909). Pamiparib binds directly to, and inhibits the activity of, PARP enzymes, preventing DNA damage repair and trapping PARP-DNA complexes at the DNA damage site [19]. In early-phase clinical studies, single-agent pamiparib demonstrated antitumor activity and was generally well tolerated in patients with advanced solid tumors [20–22]. In May 2021, data from a phase II trial (NCT03333915) supported the approval of pamiparib in China for the treatment of recurrent, advanced ovarian, fallopian tube, and primary peritoneal cancer following ≤ 2 prior lines of chemotherapy [15].

Here, we report the safety and efficacy of pamiparib in a phase II, multicenter study in Chinese patients with locally advanced or metastatic HER2− breast cancer harboring germline *BRCA1/2* mutations (g*BRCA1/2* m).

## Methods

### Study design and participants

This open-label, multicenter, phase II clinical study evaluated the efficacy and safety of pamiparib as a single-agent therapy for patients with locally advanced or metastatic TNBC or HR+/HER2− breast cancer, harboring g*BRCA1/2* m, who had progressed despite standard therapy, or for whom no standard therapy exists (Supplementary Fig. S1).

Patients were recruited from 25 sites across China. Eligible patients were adults (≥ 18 years of age) with histologically or cytologically confirmed, locally advanced or metastatic HER2− breast cancer (TNBC or HR+/HER2− breast cancer) with deleterious or suspected deleterious g*BRCA1/2* m confirmed centrally by Amoy Diagnostics Co., Ltd. in China using next-generation sequencing and multiplex ligation-dependent probe amplification methods. All patients had received ≤ 2 prior lines of chemotherapy in the locally advanced or metastatic setting. Prior platinum therapy was allowed if there was no disease progression while on treatment. Patients with HR+/HER2− breast cancer must also have received and progressed on ≥ 1 endocrine therapy in the adjuvant or metastatic setting, or have been considered medically ineligible for endocrine therapy. An Eastern Cooperative Oncology Group performance status score of 0 or 1 and measurable disease by Response Evaluation Criteria in Solid Tumors version 1.1 (RECIST v1.1) were also required. Key exclusion criteria included treatment with a systemic therapy within 14 days (or 5 half-lives, if applicable, whichever is shorter) of study drug initiation, and untreated and/or active brain metastases. Full eligibility criteria are provided in the Supplementary Appendix.

The protocol was approved by the relevant Institutional Review Board/Independent Ethics Committee for each study site. This study was carried out in accordance with the International Conference on Harmonization, Good Clinical Practice Guideline, the principles of the Declaration of Helsinki, and local laws and regulations. All patients provided written informed consent before participation in the study.

### Patient treatment and cohorts

Eligible patients were enrolled in the TNBC cohort or HR+/HER2− cohort according to estrogen receptor (ER), progesterone receptor (PR), and HER2 status determined using the most recent biopsy. Tumors expressing ER and PR, defined as ≥ 1% of tumor cells staining positive by immunohistochemistry (IHC), were considered hormone receptor-positive [23]. An IHC score of 0 or 1 + (and no evidence of in situ hybridization [ISH] amplification) or an IHC score of 2 + (and non-amplified ISH) denoted HER2−negative status [24]. All patients had deleterious or suspected deleterious g*BRCA1/2* m confirmed by central testing; mutation analyses of *BRCA1* and *BRCA2* were performed using next-generation sequencing.

All patients received pamiparib 60 mg orally twice daily (PO BID) in 28-day, continuous cycles until disease progression as assessed by investigator, unacceptable toxicity, death, withdrawal of consent, loss to follow-up, or trial termination by the sponsor. Pamiparib was administered with or without food. Dose modifications of pamiparib included dose reductions and dose interruptions; these modifications were not mutually exclusive. Treatment with pamiparib could be reduced by one dose level to 40 mg PO BID, or by two dose levels to 20 mg PO BID; a maximum of two dose reductions was permitted. Pamiparib was permitted to be withheld by the investigator for up to 28 consecutive days for medical events (or 56 days for anemia).

After treatment discontinuation, all patients were followed up for safety, survival, and further anticancer therapy. A graphical representation of the study design is available in the Supplementary Appendix (Supplementary Fig. S1).

### Endpoints and assessments

The safety analysis set consisted of all patients who received at least one dose of pamiparib. The efficacy analysis set consisted of all patients in the safety population who had measurable disease at baseline per RECIST v1.1 and had at least one evaluable post-baseline tumor assessment by independent review committee (IRC) unless they discontinued treatment due to clinical progression or death prior to tumor assessment.

The primary endpoint of this study was ORR by IRC per RECIST v1.1 in the efficacy analysis set. The key secondary endpoint was duration of response (DoR) per RECIST v1.1 by IRC and investigator. Other secondary endpoints included ORR by investigator, best overall response, disease control rate, and clinical benefit rate by IRC and investigator in the efficacy analysis set, and PFS and OS in the safety analysis set.

Blood samples collected during the pre-screening phase were analysed in a qualified central laboratory to confirm *gBRCA1/2* m status. Tumor assessments were performed once every 8 weeks (± 7 days) in the first 12 months and then once every 12 weeks (± 7 days) from year two onwards. Tumor responses were assessed separately by IRC and investigator using diagnostic-quality computed tomography or magnetic resonance imaging. Patients were followed for survival, further anticancer therapy, and diagnosis of myelodysplastic syndrome or acute myeloid leukemia approximately every 12 weeks.

Safety and tolerability were assessed throughout the study and up to 30 days after the last dose of pamiparib by monitoring the incidence and severity of treatment-emergent adverse events (TEAEs) and serious TEAEs (graded according to National Cancer Institute Common Terminology Criteria for Adverse Events [v4.03]). A TEAE was defined as an adverse event (AE) with an onset date on or after the first dose of study drug up to 30 days following treatment discontinuation. A complete list of study endpoints is provided in the Supplementary Appendix.

### Statistical analyses

This study was designed to provide adequate power to test the hypothesis for the primary endpoint of ORR by IRC in the efficacy evaluable TNBC cohort. A sample size of approximately 55 evaluable patients in the TNBC cohort was estimated to provide an alpha of 0.025 with 90% power using a binomial exact test to demonstrate a statistical difference between an estimated historical ORR of 25% with chemotherapy and an assumed ORR (by IRC) of 46% with pamiparib. The historical ORR of 25% was derived from ORRs for metastatic breast cancer with germline *BRCA* mutations after chemotherapy, and accounts for the fact that platinum-based chemotherapy, commonly used in China [25], would increase ORRs above what is observed with standard of care chemotherapy [8, 9, 26]. A sample size of approximately 20 patients was determined for the exploratory HR+/HER2− cohort. The ORR and two-sided 95% confidence interval (CI) were estimated using the binomial exact method in the efficacy analysis set. Time-to-event data, including DoR, PFS, and OS, were analyzed using the Kaplan–Meier method, with median and 95% CI estimated using the Brookmeyer and Crowley method.

A subgroup analysis of ORR by IRC was conducted in baseline characteristic subgroups and presented as a forest plot. The association of g*BRCA1*m and g*BRCA2*m with OS and PFS was explored using a Cox regression model with *BRCA* mutation type as a covariate. The hazard ratio (HR) and 95% CI were calculated separately for each cohort and the survival curve for g*BRCA1/2* m status was estimated using the Kaplan–Meier method. The incidence of AEs was summarized using descriptive statistics. All calculations and analyses were conducted using SAS version 9.4 or higher. Full statistical methods are provided in the Statistical Analysis Plan provided as a separate Supplementary File.

## Results

### Patient characteristics

Between June 2018 and April 2020, 88 patients were enrolled across 25 sites in China to receive pamiparib, 62 to the TNBC cohort and 26 to the HR+/HER2− cohort. All patients received ≥ 1 dose of pamiparib and were included in the safety analysis set. In total, there were 76 patients in the efficacy analysis set (i.e., these patients had measurable disease at baseline by IRC per RECIST v1.1 and ≥ 1 evaluable post-baseline tumor assessment [TNBC cohort, *n* = 55; HR+/HER2− cohort, *n* = 21]).

Patient demographics and baseline characteristics were representative of the target population (Table 1). The median age was 45.5 years (range: 27–67) and all patients were female (*n* = 88). The majority of patients (75.0%) presented with two or more sites of metastasis. In the TNBC cohort, *BRCA1* mutations were more common than *BRCA2* mutations (75.8% vs. 24.2%, respectively), whereas *BRCA2* mutations were more common than *BRCA1* mutations in the HR+/HER2− cohort (65.4% vs. 34.6%, respectively). Sixty patients (68.2%) had received 1 or 2 prior lines of chemotherapy in the locally advanced or metastatic setting (TNBC cohort, *n* = 45 [72.6%]; HR+/HER2− cohort, *n* = 15 [57.7%]). Almost all patients had previously been treated with anthracycline and taxane, and 42 patients (47.7%) had been previously treated with platinum-based chemotherapy (TNBC cohort, *n* = 31 [50.0%]; HR+/HER2− cohort, *n* = 11 [42.3%]). At data cutoff (October 9, 2020), the median duration of follow-up was 13.8 months (TNBC cohort, 10.9 months; HR+/HER2− cohort, 18.5 months). The median duration of treatment was 3.8 months (range: 0.7–19.4) in the TNBC cohort and 9.6 months (range: 0.9–19.4) in the HR+/HER2− cohort. Fifty-three patients remained on the study for follow-up and 10 patients remained on pamiparib treatment.

**Table 1:** Patient demographics and baseline characteristics in the safety analysis set

	TNBC cohort (*n* = 62)	HR+/HER2− cohort (*n* = 26)	Total (*N* = 88)
Median age, years (range)	45.0 (27–65)	47.5 (29–67)	45.5 (27–67)
Age group, *n* (%)			
< 50 years	43 (69.4)	16 (61.5)	59 (67.0)
50–65 years	19 (30.6)	9 (34.6)	28 (31.8)
> 65 years	0 (0.0)	1 (3.8)	1 (1.1)
Sex, *n* (%)			
Female	62 (100.0)	26 (100.0)	88 (100.0)
Race, *n* (%)			
Asian: Chinese	62 (100.0)	26 (100.0)	88 (100.0)
ECOG performance status, *n* (%)			
0	31 (50.0)	17 (65.4)	48 (54.5)
1	31 (50.0)	9 (34.6)	40 (45.5)
Number of metastatic sites, *n* (%)			
1	18 (29.0)	4 (15.4)	22 (25.0)
2	17 (27.4)	9 (34.6)	26 (29.5)
≥ 3	27 (43.5)	13 (50.0)	40 (45.5)
History of brain metastasis, *n* (%)	6 (9.7)	0 (0.0)	6 (6.8)
Visceral metastasis, *n* (%)^a^	42 (67.7)	20 (76.9)	62 (70.5)
Germline *BRCA* mutation status, *n* (%)			
*BRCA1* mutation	47 (75.8)	9 (34.6)	56 (63.6)
*BRCA2* mutation	15 (24.2)	17 (65.4)	32 (36.4)
Hormone receptor status, *n* (%)^b^			
ER positive	0 (0.0)	23 (88.5)	23 (26.1)
PR positive	0 (0.0)	19 (73.1)	19 (21.6)
Prior lines of chemotherapy, *n* (%)			
0	17 (27.4)	11 (42.3)	28 (31.8)
1	32 (51.6)	10 (38.5)	42 (47.7)
2	13 (21.0)	5 (19.2)	18 (20.5)
Prior platinum therapy, *n* (%)	31 (50.0)	11 (42.3)	42 (47.7)
Prior hormonal therapy, *n* (%)	8 (12.9)	22 (84.6)	30 (34.1)
Prior anthracycline, *n* (%)	59 (95.2)	25 (96.2)	84 (95.5)
Prior taxane, *n* (%)	60 (96.8)	26 (100.0)	86 (97.7)
Median time from initial diagnosis to study entry, years (range)	2.62 (0.3–19.9)	4.11 (1.4–11.6)	2.95 (0.3–19.9)

Data cutoff: October 9, 2020

*BRCA* breast cancer susceptibility gene, *ECOG* Eastern Cooperative Oncology Group, *ER* estrogen receptor, *HER2-* human epidermal growth factor receptor 2-negative, *HR* + hormone receptor-positive, *PR* progesterone receptor, *TNBC* triple-negative breast cancer

^a^Visceral metastasis included patients who had metastatic lesions located on the lung, liver, pleura, pleural effusion, brain, or peritoneum

^b^ER status and PR status were based on most recent biopsy

### Treatment efficacy

Efficacy results by IRC are presented by cohort (Table 2). Treatment with pamiparib demonstrated an ORR of 38.2% in the TNBC cohort and 61.9% in the HR+/HER2− cohort (TNBC cohort, 95% CI 25.4–52.3; *n* = 21 responders; HR+/HER2− cohort, 95% CI 38.4–81.9; *n* = 13 responders). ORR by investigator (Supplementary Table S1) was similar to IRC-assessed ORR (TNBC cohort, 36.4%; HR+/HER2− cohort, 57.1%).

**Table 2:** Primary and secondary efficacy endpoints in the efficacy analysis set by IRC

	TNBC cohort (*n* = 55)	HR+/HER2− cohort (*n* = 21)
Confirmed ORR, *n* (%) [95% CI]	21 (38.2) [25.4–52.3]	13 (61.9) [38.4–81.9]
Confirmed best overall response, *n* (%)		
CR	3 (5.5)	1 (4.8)
PR	18 (32.7)	12 (57.1)
SD	19 (34.5)	6 (28.6)
PD	15 (27.3)	2 (9.5)
Disease control rate (CR + PR + SD), *n* (%) [95% CI]	40 (72.7) [59.0–83.9]	19 (90.5) [69.6–98.8]
Clinical benefit rate (CR + PR + durable^a^ SD), *n* (%) [95% CI]	24 (43.6) [30.3–57.7]	15 (71.4) [47.8–88.7]
DoR		
Events, *n* (%)	11 (20.0)	10 (47.6)
Median, months (95% CI)^b^	7.0 (3.9–NE)	7.5 (5.6–14.8)

Data cutoff: October 9, 2020

*CI* confidence interval, *CR* complete response, *DoR* duration of response, *HER2-* human epidermal growth factor receptor 2-negative, *HR* + hormone receptor-positive, *IRC* independent review committee, *NE* not estimable, *ORR* objective response rate, *PD* progressive disease, *PR* partial response, *SD* stable disease, *TNBC* triple-negative breast cancer

^a^Durable SD was defined as lasting ≥ 24 weeks

^b^Medians were estimated by the Kaplan–Meier method, with 95% CIs estimated using the method of Brookmeyer and Crowley

**Fig. 1:**
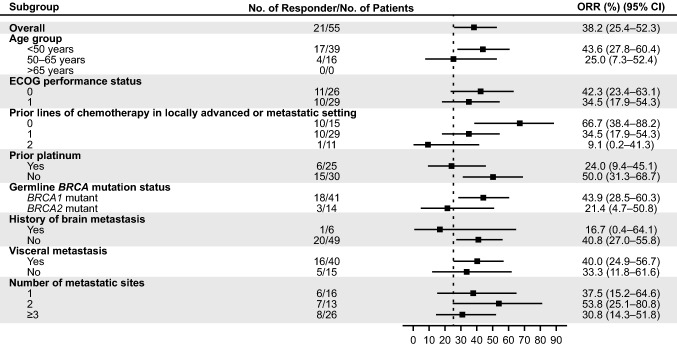
Subgroup analysis: ORR by IRC in the TNBC cohort in the efficacy analysis set. The two-sided 95% CI was calculated using the binomial exact method. The historical ORR with chemotherapy in a similar population is represented by a dashed line on the figure and was estimated to be 25%. Data cutoff: October 9, 2020. *BRCA* breast cancer susceptibility gene, *CI* confidence interval, *ECOG* Eastern Cooperative Oncology Group, *IRC* independent review committee, *ORR* objective response rate, *TNBC* triple-negative breast cancer

**Fig. 2:**
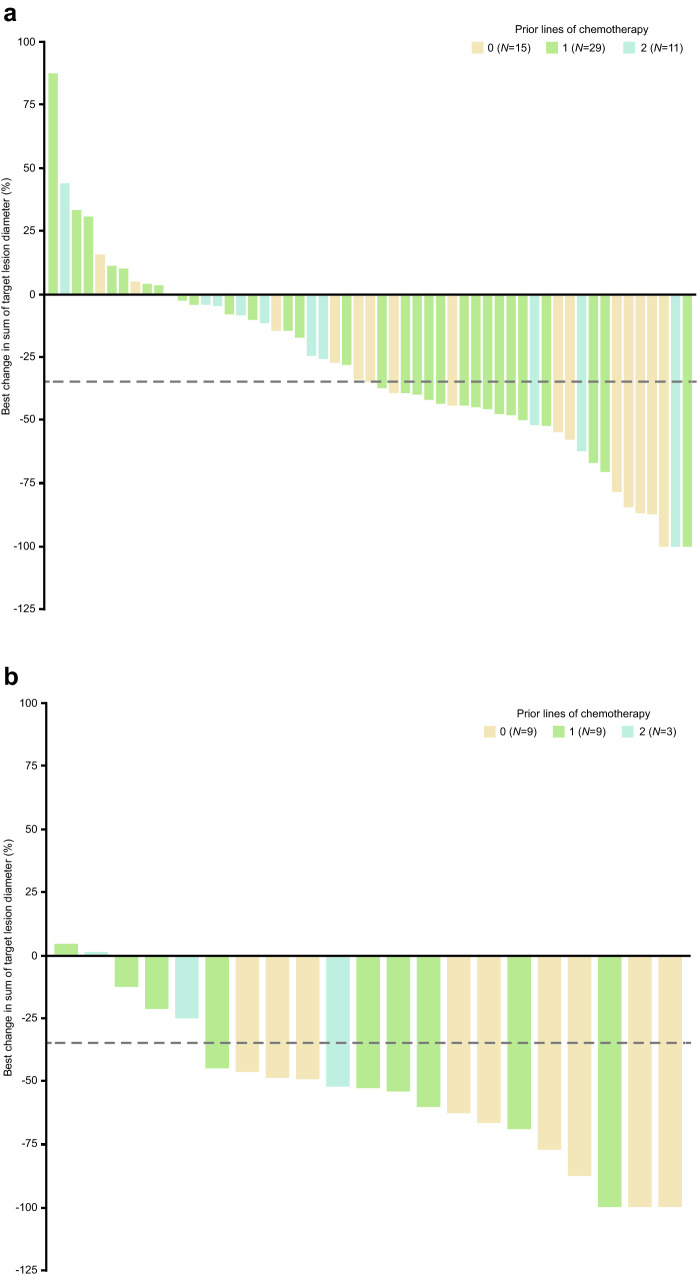
Best percentage change in target lesion diameter by IRC in patients in the **a** TNBC cohort and **b** HR+/HER2− cohort, grouped by prior lines of chemotherapy in the efficacy analysis set. *HER2-* human epidermal growth factor receptor 2-negative, *HR* + hormone receptor-positive, *IRC* independent review committee, *TNBC* triple-negative breast cancer

In both cohorts, subgroup analyses of ORR showed that response rates by IRC were higher in patients with fewer lines of prior chemotherapy (TNBC cohort, no prior lines, *n* = 10/15 [66.7%]; 1 prior line, *n* = 10/29 [34.5%]; 2 prior lines, *n* = 1/11 [9.1%] (Fig. 1); HR+/HER2− cohort, no prior lines, *n* = 8/9 [88.9%]; 1 prior line, *n* = 4/9 [44.4%]; 2 prior lines, *n* = 1/3 [33.3%]) (Supplementary Fig. S2). A similar pattern was observed in the best percentage change from baseline in target lesion diameter presented in Fig. 2a and b.

In the TNBC cohort, response rates by IRC were higher in patients who were platinum-naïve (*n* = 15/30 [50.0%]) compared with patients who had received prior platinum therapy (*n* = 6/25 [24.0%]) (Fig. 1). This was also observed in the HR+/HER2− cohort, where ORR in platinum-naïve patients was 75.0% (*n* = 9/12) versus 44.4% in platinum-treated patients (*n* = 4/9) (Supplementary Fig. S2).

In the TNBC cohort, median DoR by IRC in the efficacy analysis set was 7.0 months (95% CI 3.9–not estimable [NE]) (Table 2). In the HR+/HER2− cohort, median DoR was 7.5 months (95% CI 5.6–14.8) (Table 2). Compared with IRC-assessed DoR, median DoR by investigator was numerically shorter for the TNBC cohort (5.6 months) but comparable for the HR+/HER2− cohort (7.6 months) (Supplementary Table S1). Median time to response by IRC was 1.8 months (range: 1.7–4.7) in the TNBC cohort and 1.9 months (range: 1.7–7.3) in the HR+/HER2− cohort.

**Fig. 3:**
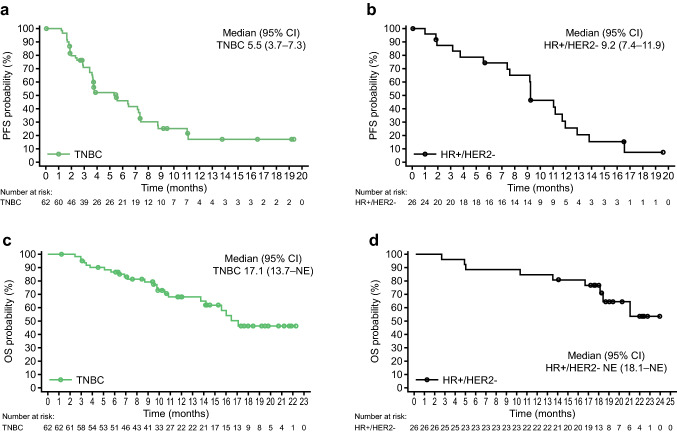
PFS by IRC in the **a** TNBC cohort and **b** HR+/HER2− cohort, and OS in the **c** TNBC cohort and **d** HR+/HER2− cohort in the safety analysis set. Median PFS and OS were estimated by the Kaplan–Meier method, with 95% CIs estimated using the method of Brookmeyer and Crowley. *CI* confidence interval, *HER2−* human epidermal growth factor receptor 2-negative, *HR*+ hormone receptor-positive, *IRC* independent review committee, *NE* not estimable, *OS* overall survival, *PFS* progression-free survival, *TNBC* triple-negative breast cancer

In the TNBC cohort, median PFS by IRC in the safety analysis set was 5.5 months (95% CI 3.7–7.3) (Fig. 3a). Five patients remained on treatment for over one year at data cutoff. In the HR+/HER2− cohort, median PFS was 9.2 months (95% CI 7.4–11.9) (Fig. 3b). Median PFS by investigator is presented in Supplementary Fig. S3. Median OS in the safety analysis set was 17.1 months (95% CI 13.7–NE) in the TNBC cohort and NE in the HR+/HER2− cohort (95% CI 18.1–NE) (Fig. 3c and d).

Germline *BRCA1*m were more prevalent than g*BRCA2*m in the TNBC cohort (Table 1); however, no differences in OS and PFS by g*BRCAm* status were observed (Supplementary Fig. S4a and c). In the HR+/HER2− cohort, g*BRCA2*m were more prevalent than g*BRCA1*m (Table 1) and were associated with significantly longer PFS (11.7 months vs. 3.7 months, respectively; HR 0.114 [95% CI 0.032–0.403]) but there was no significant difference in OS (NE for both mutations) (Supplementary Fig. S4b and d).

### Treatment safety

TEAEs and treatment-related TEAEs (trTEAEs) are summarized in Tables 3 and 4. TEAEs that were Grade 3 or higher (≥ Grade 3) were experienced by 54 (61.4%) patients and ≥ Grade 3 trTEAEs were experienced by 53 (60.2%) patients. Serious TEAEs were experienced by 19 (21.6%) patients (Table 3). The most common all-grade and ≥ Grade 3 TEAEs and trTEAEs were hematologic events including anemia, decreased neutrophil count, and decreased white blood cell count (Table 4). No cases of myelodysplastic syndrome or acute myeloid leukemia were reported.

**Table 3:** Overall summary of TEAEs and trTEAEs in the safety analysis set

*n* (%)	TNBC cohort (*n* = 62)	HR+/HER2− cohort (*n* = 26)	Total (*N* = 88)
Patients with at least one TEAE	61 (98.4)	26 (100.0)	87 (98.9)
≥ Grade 3	37 (59.7)	17 (65.4)	54 (61.4)
Serious	12 (19.4)	7 (26.9)	19 (21.6)
Leading to death	1 (1.6)	0 (0.0)	1 (1.1)
Leading to treatment discontinuation	1 (1.6)	1 (3.8)	2 (2.3)
Leading to dose modification	45 (72.6)	21 (80.8)	66 (75.0)
Leading to dose interruption	42 (67.7)	21 (80.8)	63 (71.6)
Leading to dose reduction	40 (64.5)	17 (65.4)	57 (64.8)
Patients with at least one trTEAE	61 (98.4)	26 (100.0)	87 (98.9)
≥ Grade 3	36 (58.1)	17 (65.4)	53 (60.2)
Serious	9 (14.5)	6 (23.1)	15 (17.0)
Leading to death	0 (0.0)	0 (0.0)	0 (0.0)
Leading to treatment discontinuation	1 (1.6)	1 (3.8)	2 (2.3)
Leading to dose modification	45 (72.6)	20 (76.9)	65 (73.9)
Leading to dose interruption	42 (67.7)	20 (76.9)	62 (70.5)
Leading to dose reduction	40 (64.5)	17 (65.4)	57 (64.8)

AEs were graded according to NCI CTCAE (v4.03). A patient with multiple AEs under a system organ class and preferred term was counted only once for the system organ class and preferred term, under the maximum severity category. Data cutoff: October 9, 2020

*AE* adverse event, *HER2-* human epidermal growth factor receptor 2-negative, *HR* + hormone receptor-positive, *NCI CTCAE* National Cancer Institute Common Terminology Criteria for Adverse Events, *TEAE* treatment-emergent adverse event, *trTEAE* treatment-related treatment-emergent adverse event, *TNBC* triple-negative breast cancer

**Table 4:** Most common all-grade and ≥ Grade 3 TEAEs and trTEAEs occurring in ≥ 10% of patients (for all grades in any cohort) in the safety analysis set

*n* (%)	TNBC cohort (*n* = 62)		HR+/HER2− cohort (*n* = 26)		Total (*N* = 88)	
	All-grades	≥ Grade 3	All-grades	≥ Grade 3	All-grades	≥ Grade 3
Hematologic TEAEs						
Anemia	51 (82.3)	22 (35.5)	26 (100.0)	13 (50.0)	77 (87.5)	35 (39.8)
White blood cell count decreased	35 (56.5)	12 (19.4)	21 (80.8)	7 (26.9)	56 (63.6)	19 (21.6)
Neutrophil count decreased	34 (54.8)	18 (29.0)	18 (69.2)	8 (30.8)	52 (59.1)	26 (29.5)
Platelet count decreased	18 (29.0)	5 (8.1)	10 (38.5)	3 (11.5)	28 (31.8)	8 (9.1)
Leukopenia	8 (12.9)	3 (4.8)	6 (23.1)	2 (7.7)	14 (15.9)	5 (5.7)
Neutropenia	6 (9.7)	3 (4.8)	5 (19.2)	2 (7.7)	11 (12.5)	5 (5.7)
Non-hematologic TEAEs						
Nausea	29 (46.8)	1 (1.6)	15 (57.7)	0 (0.0)	44 (50.0)	1 (1.1)
Vomiting	22 (35.5)	1 (1.6)	14 (53.8)	0 (0.0)	36 (40.9)	1 (1.1)
Decreased appetite	25 (40.3)	0 (0.0)	8 (30.8)	0 (0.0)	33 (37.5)	0 (0.0)
Alanine aminotransferase increased	13 (21.0)	0 (0.0)	10 (38.5)	0 (0.0)	23 (26.1)	0 (0.0)
Diarrhea	11 (17.7)	0 (0.0)	7 (26.9)	0 (0.0)	18 (20.5)	0 (0.0)
Aspartate aminotransferase increased	10 (16.1)	1 (1.6)	7 (26.9)	0 (0.0)	17 (19.3)	1 (1.1)
Blood bilirubin increased	8 (12.9)	0 (0.0)	7 (26.9)	0 (0.0)	15 (17.0)	0 (0.0)
Malaise	11 (17.7)	0 (0.0)	4 (15.4)	0 (0.0)	15 (17.0)	0 (0.0)
Asthenia	9 (14.5)	1 (1.6)	4 (15.4)	0 (0.0)	13 (14.8)	1 (1.1)
Upper respiratory tract infection	6 (9.7)	0 (0.0)	7 (26.9)	1 (3.8)	13 (14.8)	1 (1.1)
Weight decreased	11 (17.7)	0 (0.0)	1 (3.8)	0 (0.0)	12 (13.6)	0 (0.0)
Cough	6 (9.7)	0 (0.0)	4 (15.4)	0 (0.0)	10 (11.4)	0 (0.0)
Insomnia	6 (9.7)	0 (0.0)	4 (15.4)	0 (0.0)	10 (11.4)	0 (0.0)
Dizziness	6 (9.7)	0 (0.0)	3 (11.5)	0 (0.0)	9 (10.2)	0 (0.0)
Hematologic trTEAEs						
Anemia	51 (82.3)	22 (35.5)	26 (100.0)	13 (50.0)	77 (87.5)	35 (39.8)
White blood cell count decreased	35 (56.5)	12 (19.4)	20 (76.9)	7 (26.9)	55 (62.5)	19 (21.6)
Neutrophil count decreased	34 (54.8)	18 (29.0)	17 (65.4)	8 (30.8)	51 (58.0)	26 (29.5)
Platelet count decreased	18 (29.0)	5 (8.1)	10 (38.5)	3 (11.5)	28 (31.8)	8 (9.1)
Leukopenia	8 (12.9)	3 (4.8)	6 (23.1)	2 (7.7)	14 (15.9)	5 (5.7)
Neutropenia	6 (9.7)	3 (4.8)	5 (19.2)	2 (7.7)	11 (12.5)	5 (5.7)
Non-hematologic trTEAEs						
Nausea	28 (45.2)	1 (1.6)	15 (57.7)	0 (0.0)	43 (48.9)	1 (1.1)
Vomiting	21 (33.9)	1 (1.6)	12 (46.2)	0 (0.0)	33 (37.5)	1 (1.1)
Decreased appetite	23 (37.1)	0 (0.0)	8 (30.8)	0 (0.0)	31 (35.2)	0 (0.0)
Alanine aminotransferase increased	10 (16.1)	0 (0.0)	9 (34.6)	0 (0.0)	19 (21.6)	0 (0.0)
Diarrhea	11 (17.7)	0 (0.0)	6 (23.1)	0 (0.0)	17 (19.3)	0 (0.0)
Aspartate aminotransferase increased	10 (16.1)	1 (1.6)	6 (23.1)	0 (0.0)	16 (18.2)	1 (1.1)
Malaise	11 (17.7)	0 (0.0)	4 (15.4)	0 (0.0)	15 (17.0)	0 (0.0)
Blood bilirubin increased	7 (11.3)	0 (0.0)	7 (26.9)	0 (0.0)	14 (15.9)	0 (0.0)
Weight decreased	11 (17.7)	0 (0.0)	1 (3.8)	0 (0.0)	12 (13.6)	0 (0.0)
Asthenia	8 (12.9)	1 (1.6)	4 (15.4)	0 (0.0)	12 (13.6)	1 (1.1)

AEs were graded according to NCI CTCAE (v4.03). A patient with multiple AEs under a system organ class and preferred term was counted only once for the system organ class and preferred term, under the maximum severity category. Data cutoff: October 9, 2020

*AE* adverse event, *HER2-* human epidermal growth factor receptor 2-negative, *HR* + hormone receptor-positive, *NCI CTCAE* National Cancer Institute Common Terminology Criteria for Adverse Events, *TEAE* treatment-emergent adverse event, *trTEAE* treatment-related treatment-emergent adverse event, *TNBC* triple-negative breast cancer

AEs were generally manageable through supportive care or dose modification. TEAEs led to dose modification in 66 patients (75.0%), of whom 63 (71.6%) had their dose interrupted and 57 (64.8%) had their dose reduced (Table 3). In total, 59.1% of patients had a single dose reduction. Treatment discontinuation due to TEAEs was rare and occurred for one patient (1.6%) in the TNBC cohort and one patient (3.8%) in the HR+/HER2− cohort. One TEAE (1.1%) leading to death occurred in the TNBC cohort and was considered by the investigator as not likely to be related to the trial drug. No trTEAEs leading to death were reported.

## Discussion

In this phase II, open-label study, treatment with pamiparib demonstrated encouraging and durable clinical activity in patients with locally advanced or metastatic HER2− breast cancer harboring g*BRCA1/2* m. The ORR of 38.2% in the TNBC cohort was significantly higher than the predefined historical ORR of 25% for chemotherapy (*P* = 0.0210) [8, 9], and higher than the pooled ORRs for first- or later-line standard chemotherapy regimens (23% and 11%, respectively) [6]. The ORR of 61.9% in the HR+/HER2− cohort is promising. A median DoR of 7.0 months was observed in the TNBC cohort and 7.5 months in the HR+/HER2− cohort, indicating a durable response. These efficacy results are comparable to those observed in other studies that investigated PARPi for second- or third-line treatment in patients with HER2− advanced or metastatic breast cancer harboring g*BRCA1/2* m [8, 9, 27].

A higher percentage of patients in this study received prior platinum-based chemotherapy compared with the OlympiAD and EMBRACA studies [8, 9]. This was an expected observation given that platinum-based chemotherapy is one of the preferred first-line treatment options for advanced breast cancer in China [25]. In this study, a trend towards a higher ORR was observed in patients who had received fewer prior lines of chemotherapy or were platinum-naïve, regardless of disease subtype. A similar trend was observed in EMBRACA, whereby both platinum-naïve and platinum-treated patients experienced clinical benefit from treatment with talazoparib, the benefit was greater in patients with no prior platinum exposure [9, 28]. Similarly, in the ABRAZO study, response to talazoparib was poorer in patients with shorter platinum-free intervals compared with longer platinum-free intervals [27].

The distribution of g*BRCA1* and g*BRCA2* mutations across the two cohorts was performed; however, the interpretation of these data must be treated with caution due to the small sample size of each subgroup. The incidence of *BRCA1* and *BRCA2* mutations across the two cohorts was consistent with known associations; the *BRCA1* mutation is known to associate preferentially with TNBC, and *BRCA2* mutation with HR+/HER2− disease [29, 30]. PFS in patients with g*BRCA1*m was numerically longer in the TNBC cohort compared with the HR+/HER2− cohort (median PFS: TNBC, 5.6 months; HR+/HER2−, 3.7 months). This is generally consistent with other PARPi clinical trial data in patients with breast cancer [8, 9].

The safety profile of pamiparib was considered acceptable and was consistent with the overall safety profile of pamiparib in previous studies, and the safety profile of other PARPi [8, 9, 18, 21, 22, 27]. In this study, hematologic AEs including anemia, decreased neutrophil count, and decreased white blood cell count were the most common TEAEs. However, the use of a dose-modification protocol managed these hematologic events appropriately through dose interruption and/or dose reduction, together with supportive care per local treatment guidance. The use of this protocol led to more dose reductions than expected. Although hematologic events including anemia, decreased neutrophil count, and decreased white blood cell count were the most frequently occurring ≥ Grade 3 TEAEs, no neutropenic sepsis or neutropenic infection events were reported, and no significant hemorrhage events occurred as a consequence of decreased white blood cell count. Importantly, no cases of myelodysplastic syndrome or acute myeloid leukemia were observed, despite secondary hematologic malignancies being recognized as a rare AE associated with PARPi [31–33].

To the best of our knowledge, this is one of the largest studies conducted in Chinese women with locally advanced or metastatic HER2− breast cancer harboring g*BRCA1/2* m. The strength of this study is that it helps to address the lack of PARPi data for advanced or metastatic breast cancer in China, where PARPi have not been approved, and only one study to date has reported efficacy and safety results for PARPi in this patient population [18]. The open-label, single-arm nature of this study may have resulted in subjectivity and variability between investigators in their assessment of tumor response. However, the prospective use of an IRC to assess tumor response minimizes this potential bias. Recruiting patients with g*BRCA1/2 m* was challenging due to a low prevalence in this patient population, and the sample size of this study is relatively small. Nevertheless, the sample size of the TNBC cohort provided adequate power to test the study hypothesis. This was a single-arm study and, consequently, the lack of head-to-head studies versus best available treatment in breast cancer is a limitation. However, data from this study suggest that, with the caveat of cross-trial comparison, the efficacy and safety profile of pamiparib appears comparable with those observed in trials of other PARPi in patients with HER2− breast cancer harboring g*BRCA1/2* m [8, 9, 27], including patients from Asia [18].

## Conclusion

Pamiparib demonstrated promising clinical activity in patients with locally advanced or metastatic HER2 −  breast cancer harboring g*BRCA1*/*2* m, with an acceptable safety profile that was generally consistent with therapies in the same class.

## Supplementary Information

Below is the link to the electronic supplementary material.

Supplementary file1 (DOCX 541 KB)

## Data Availability

On request, and subject to certain criteria, conditions, and exceptions, BeiGene, Ltd., will provide access to individual de-identified participant data from BeiGene-sponsored global interventional clinical studies conducted for medicines (1) for indications that have been approved or (2) in programs that have been terminated. BeiGene will also consider requests for the protocol, data dictionary, and statistical analysis plan. Data requests may be submitted to DataDisclosure@beigene.com.
