# Supplementary material for: Pamiparib in patients with locally advanced or metastatic HER2-negative breast cancer with germline BRCA mutations: a phase II study
Source: Breast Cancer Res Treat. 2022 Dec 2;197(3):489–501. doi: 10.1007/s10549-022-06785-z (PMC9883365; doi:10.1007/s10549-022-06785-z)
Supplement: Supplementary file 1 — Supplementary file1 (DOCX 541 KB) [file 10549_2022_6785_MOESM1_ESM.docx]

# **Supplementary Appendix for:**

Pamiparib in patients with locally advanced or metastatic HER2-negative breast cancer with germline *BRCA* mutations: a phase II study

Binghe Xu, Tao Sun, Yanxia Shi, Jiuwei Cui, Yongmei Yin, Quchang Ouyang, Qiang Liu, Qingyuan Zhang, Yiding Chen, Shouman Wang, Xiaojia Wang, Zhongsheng Tong, Yahua Zhong, Jiayu Wang, Min Yan, Xi Yan, Chuan Wang, Jifeng Feng, Xiuli Wang, Gang Hu, Ying Cheng, Ruimin Ge, Zhaoyin Zhu, Wa Zhang, Zhimin Shao

Corresponding author:

Binghe Xu

National Cancer Center, Cancer Institute and Hospital, Chinese Academy of Medical Sciences and Peking Union Medical College, Beijing, China

Email: xubinghe@csco.org.cn

*Breast Cancer Research and Treatment*

**Supplement 1 (online only)**

**Investigators List (NCT03575065)**

| **Country** | **Principal Investigator** |
| --- | --- |
| China | Chen, Yiding |
| China | Cheng, Ying |
| China | Cui, Jiuwei |
| China | Du, Xiuping |
| China | Feng, Jifeng |
| China | Hu, Gang |
| China | Liu, Dequan |
| China | Liu, Qiang |
| China | Ouyang, Quchang |
| China | Shao, Zhimin |
| China | Shi, Yanxia |
| China | Sun, Tao |
| China | Tong, Zhongsheng |
| China | Wang, Chuan |
| China | Wang, Shouman |
| China | Wang, Shu |
| China | Wang, Xiaojia |
| China | Wang, Xiuli |
| China | Wang, Yongsheng |
| China | Xu, Binghe |
| China | Yan, Min |
| China | Yan, Xi |
| China | Yin, Yongmei |
| China | Zhang, Qingyuan |
| China | Zhong, Yahua |

**Complete study protocol**

BeiGene BGB-290-201 CLINICAL STUDY PROTOCOL Protocol Amendment 1.0 30 October 2018

**Statistical Analysis Plan**

BeiGene, Ltd. BGB-290-201 (Statistical Analysis Plan) October 13, 2020 Version 1.0

**Full inclusion and exclusion criteria**

*Inclusion criteria*

Patients may be enrolled in the study only if they meet all of the following criteria:

1. Signed informed consent form (ICF)
2. Age ≥18 years on day of signed ICF
3. Confirmed deleterious or suspected deleterious germline *BRCA1* or *BRCA2* mutation
   - Germline *BRCA1/2* mutation testing in central laboratory prior to Day 1 of Cycle 1
4. Locally advanced or metastatic breast cancer despite standard therapy and the following:
   - Histologically or cytologically confirmed human epidermal growth factor receptor 2-negative (HER2-) breast cancer (triple-negative breast cancer or estrogen receptor-positive and/or progesterone receptor-positive)
   - ≤2 prior lines of chemotherapy in advanced or metastatic setting
   - Prior platinum therapy allowed as long as no disease progression while on treatment, or if given in neoadjuvant/adjuvant setting with ≥6 months from last platinum to relapse
   - Prior therapy with an anthracycline and/or a taxane in neoadjuvant/adjuvant or metastatic setting
   - Archival tumor tissues will be collected from all patients, if available
   - For hormone receptor-positive/HER2- breast cancer only: patients must have received and progressed on at least one endocrine therapy either in adjuvant or metastatic setting, or have disease that the treating physician believes to be inappropriate for endocrine therapy
5. Measurable disease as defined per Response Evaluation Criteria in Solid Tumors (RECIST) version 1.1
   - Tumor lesions situated in a previously irradiated area, or in an area subjected to other loco-regional therapy, are usually not considered measurable unless there has been demonstrated progression in the lesion.
6. Eastern Cooperative Oncology Group performance status ≤1 (Study Protocol, Appendix 3)
7. Ability to swallow whole capsules
8. Ability to comply with study requirements independently
9. Adequate hematologic and organ function as defined by the following laboratory values (obtained ≤14 days before Day 1 of Cycle 1):
   - Absolute neutrophil count ≥1500/mL or ≥1.5 × 10^9^/L
   - Platelet count ≥75,000/mL or ≥75 × 10^9^/L
   - Hemoglobin ≥9 g/dL or ≥90 g/L (≥14 days after growth factor support or transfusion)
   - Estimated glomerular filtration rate ≥30 mL/min/1.73m^2^ by Chronic Kidney Disease Epidemiology Collaboration equation (Study Protocol, Appendix 4)
   - Total serum bilirubin ≤1.5 × upper limit of normal (ULN)
   - Aspartate aminotransferase (AST) and alanine aminotransferase (ALT) ≤3 × ULN, if liver function abnormalities are due to liver metastasis, then AST and ALT ≤5 × ULN 10
10. For females of childbearing potential and nonsterile males, must practice highly effective methods of birth control for the duration of the study and for at least 6 months after last study drug

*Exclusion criteria*

Patients will not be enrolled in the study for any of the following reasons:

1. Unresolved acute effects of prior therapy of ≥Grade 2
   - Except for AEs not considered a likely safety risk (e.g., alopecia, neuropathy, and specific laboratory abnormalities)
2. Prior treatment with a poly (ADP-ribose) polymerase (PARP) inhibitor
   - Subtherapeutic exposure to a PARP inhibitor for ≤28 days is permissible provided it was not the most recent prior therapy
3. Chemotherapy, hormonal therapy, radiotherapy, biologic therapy, immunotherapy, investigational agent, anticancer Chinese medicine, or anticancer herbal remedies ≤14 days (or ≤5 half-lives, if applicable, whichever is shorter) prior to Day 1 of
   Cycle 1
   - Bisphosphonate and receptor activator of nuclear factor kappa-Β ligand inhibitors are allowed for bone metastases if initiated before enrollment and at a stable dose
4. Major surgical procedure, open biopsy, or significant traumatic injury ≤14 days prior to Day 1 of Cycle 1, or anticipation of need for major surgical procedure during the course of the study
   - Placement of vascular access device is not considered major surgery
5. Diagnosis of myelodysplastic syndrome
6. Other diagnosis of malignancy
   - Except for surgically excised nonmelanoma skin cancer, adequately treated carcinoma in situ of the cervix, localized prostate cancer treated with curative intent, adequately treated low-stage bladder cancer, ductal carcinoma in situ treated surgically with curative intent, or a malignancy diagnosed ≥5 years ago with no current evidence of disease and no therapy ≥5 years prior to Day 1 of Cycle 1
7. Untreated and/or active brain metastases
   - A scan to confirm the absence of brain metastases is not required
   - Patients with treated brain metastases must be off corticosteroids for ≥2 weeks and have no signs or symptoms of progressive brain metastases
8. Active infection requiring systemic treatment, active viral hepatitis, or active tuberculosis
9. Any of the following cardiovascular criteria:
   - Cardiac chest pain, defined as moderate pain that limits instrumental activities of daily living, ≤28 days prior to Day 1 of Cycle 1
   - Symptomatic pulmonary embolism ≤28 days prior to Day 1 of Cycle 1
   - Any history of acute myocardial infarction ≤6 months prior to Day 1 of Cycle 1
   - Any history of heart failure meeting New York Heart Association Classification III or IV (see Study Protocol, Appendix 9) ≤6 months prior to Day 1 of Cycle 1
   - Any event of ventricular arrhythmia ≥Grade 2 in severity ≤6 months prior to Day 1 of Cycle 1
   - Any history of cerebral vascular accident ≤6 months prior to Day 1 of Cycle 1
10. Previous complete gastric resection, chronic diarrhea, active inflammatory gastrointestinal disease, or any other disease-causing malabsorption syndrome
    - Gastroesophageal reflux disease under treatment with proton pump inhibitors is allowed
11. Active bleeding disorder, including gastrointestinal bleeding, as evidenced by hematemesis, significant hemoptysis, or melena ≤6 months prior to Day 1 of Cycle 1
12. Use ≤10 days (or ≤5 half-lives, whichever is shorter), prior to Day 1 of Cycle 1, or anticipated need for food or drugs known to be strong or moderate CYP3A inhibitors or strong CYP3A inducers (Study Protocol, Appendix 6)
13. Pregnancy or nursing
    - Females of childbearing potential require a negative urine or serum pregnancy test ≤7 days prior to Day 1 of Cycle 1
14. Significant intercurrent illness that may result in the patient’s death before death from breast cancer
15. Known history of intolerance to the excipients of the pamiparib capsule

**Complete list and definitions of study endpoints**

*Primary endpoint*

- Objective response rate (ORR) - defined as the proportion of patients who achieved a best overall response of complete response (CR) or partial response (PR), assessed by independent review committee (IRC) per RECIST v1.1

*Secondary endpoints*

- ORR - defined as the proportion of patients who achieved a best overall response of CR or PR, assessed by investigator per RECIST v1.1
- Progression-free survival - defined as the time from first dose of pamiparib to the first documented disease progression assessed by IRC and investigator per RECIST v1.1 or death due to any cause
- Duration of response - defined as the time from first determination of a confirmed best overall response until the first documentation of progression or death, whichever comes first, assessed by IRC and investigator per RECIST v1.1
- Best overall response (BOR) - defined as the BOR recorded from the start of the treatment until data cutoff or start of new antineoplastic treatment, assessed by IRC and investigator per RECIST v1.1
- Disease control rate - defined as the proportion of patients who achieved a confirmed BOR of CR, PR, or stable disease (SD) assessed by IRC and investigator per RECIST v1.1
- Clinical benefit rate - defined as proportion of patients with confirmed CR or confirmed PR or a durable SD (lasting ≥24 weeks) assessed by IRC and investigator per RECIST v1.1
- Overall survival - defined as time from the first dose of pamiparib to the date of death due to any cause
- Incidence, timing, and severity of treatment-emergent adverse events graded according to the National Cancer Institute Common Terminology Criteria for Adverse Events, version 4.03 (Common Toxicity Criteria Version 4.03) or higher; changes in vital signs, physical findings, and clinical laboratory results

**Table S1** Secondary efficacy endpoints in the efficacy analysis set by investigator

|  | **TNBC cohort**  **(*n*=55)** | **HR+/HER2- cohort**  **(*n*=21)** |
| --- | --- | --- |
| Confirmed ORR,  *n* (%) [95% CI] | 20 (36.4) [23.8–50.4] | 12 (57.1) [34.0–78.2] |
| Confirmed best overall response, *n* (%) |  |  |
| CR | 2 (3.6) | 0 (0.0) |
| PR | 18 (32.7) | 12 (57.1) |
| SD | 20 (36.4) | 5 (23.8) |
| PD | 15 (27.3) | 4 (19.0) |
| Disease control rate (CR + PR + SD), *n* (%) [95% CI] | 40 (72.7) [59.0–83.9] | 17 (81.0) [58.1–94.6] |
| Clinical benefit rate (CR + PR + durable^a^ SD), *n* (%) [95% CI] | 23 (41.8) [28.7–55.9] | 14 (66.7) [43.0–85.4] |
| DoR |  |  |
| Events, *n* (%) | 14 (25.5) | 11 (52.4) |
| Median, months (95% CI) ^b^ | 5.6 (4.6–13.0) | 7.6 (6.0–13.9) |

Data cutoff: October 9, 2020

*CI* confidence interval, *CR* complete response, *DoR* duration of response, *HER2-* human epidermal growth factor receptor 2-negative, *HR+* hormone receptor-positive, *ORR* overall response rate, *PD* progressive disease, *PR* partial response, *SD* stable disease, *TNBC* triple-negative breast cancer

^a^Durable SD was defined as lasting ≥24 weeks

^b^Medians were estimated by the Kaplan-Meier method, with 95% CIs estimated using the method of Brookmeyer and Crowley

**Fig. S1** Study design


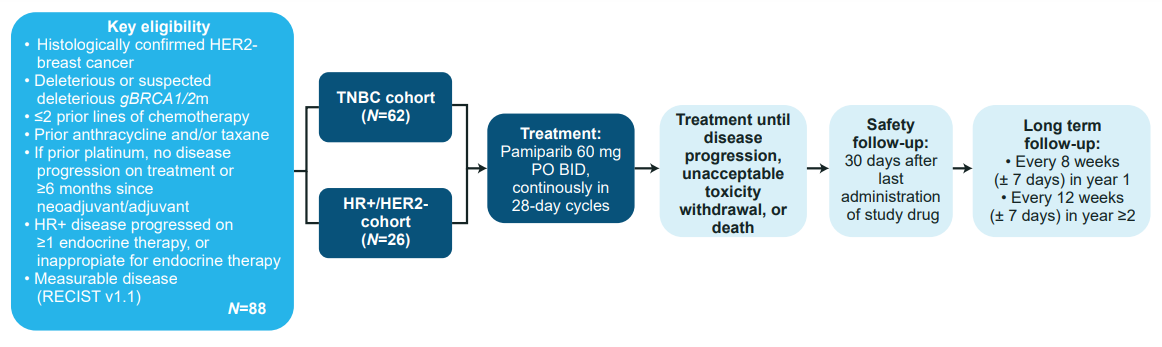


*BID* twice daily, *gBRCA1/2m* germline breast cancer susceptibility gene 1/2 mutation, *HER2-* human epidermal growth factor receptor 2-negative, *HR+* hormone receptor-positive, *PO* oral, *RECIST* Response Evaluation Criteria in Solid Tumors, *TNBC* triple-negative breast cancer

**Fig. S2** Subgroup analysis: ORR by IRC in the HR+/HER2- cohort in the efficacy analysis set
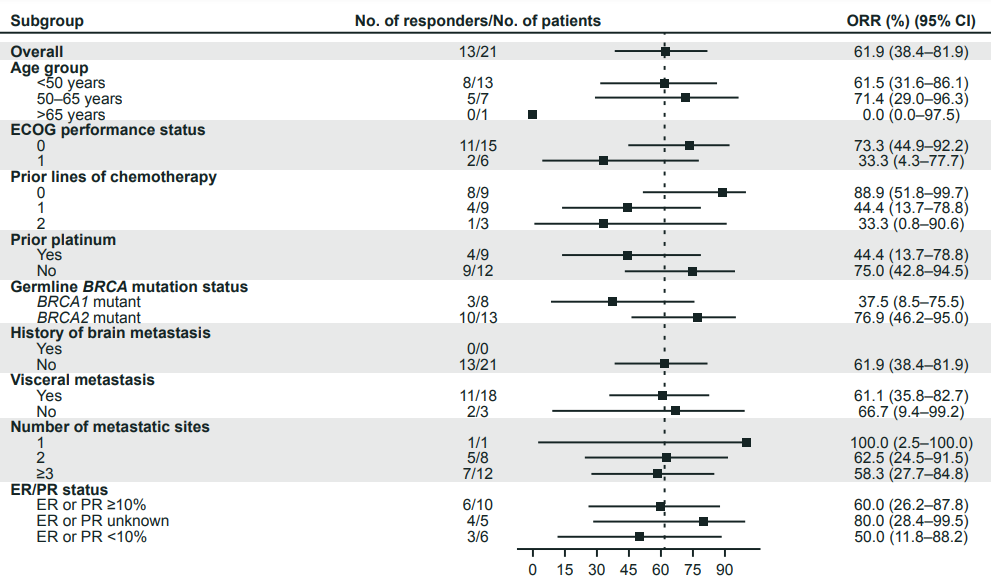


The two-sided 95% CI was calculated using the binomial exact method. Data cutoff: October 9, 2020

*BRCA* breast cancer susceptibility gene, *CI* confidence interval, *ECOG* Eastern Cooperative Oncology Group, *ER* estrogen receptor, *HER2-* human epidermal growth factor receptor 2-negative, *HR+* hormone receptor-positive, *IRC* independent review committee, *ORR* overall response rate, *PR* progesterone receptor

**Fig. S3** PFS by investigator in the (**a**) TNBC cohort and (**b**) HR+/HER2- cohort in the safety analysis set


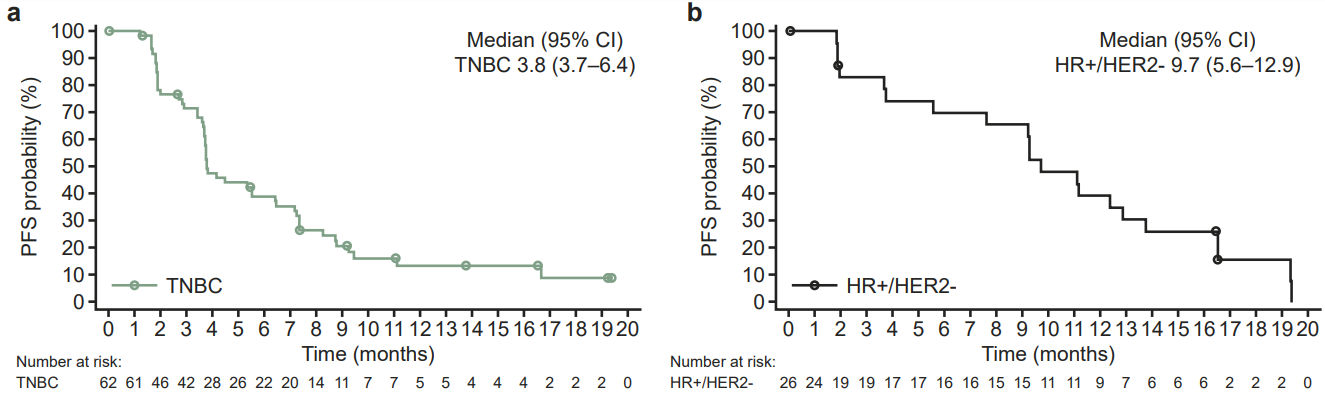


Median PFS was estimated by the Kaplan-Meier method, with 95% CIs estimated using the method of Brookmeyer and Crowley

*CI* confidence interval, *HER2-* human epidermal growth factor receptor 2-negative, *HR+* hormone receptor-positive, *PFS* progression-free survival, *TNBC* triple-negative breast cancer

**Fig. S4** OS by g*BRCA*m status in the (**a**) TNBC cohort and (**b**) HR+/HER2- cohort, and PFS by g*BRCA*m status in the (**c**) TNBC cohort and (**d**) HR+/HER2- cohort in the safety analysis set


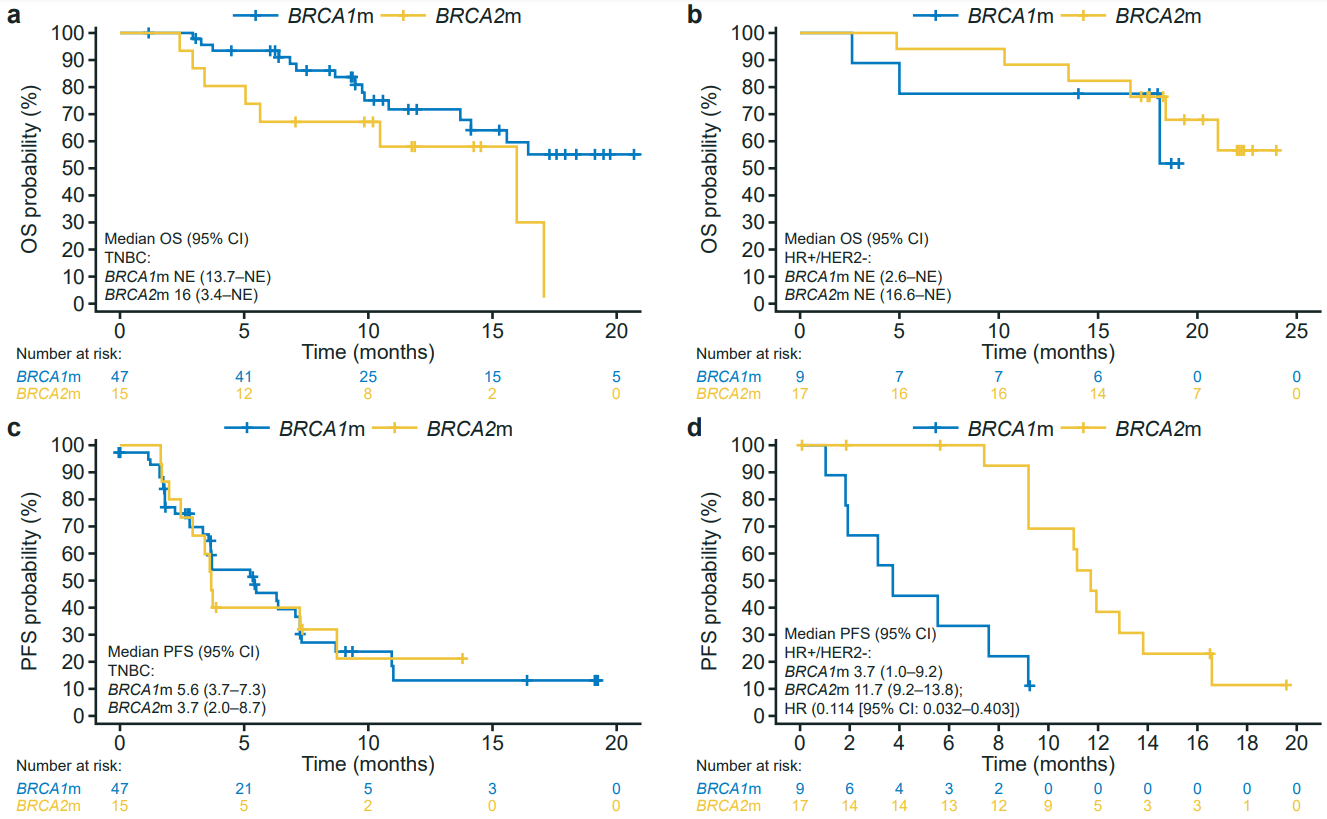


The association of *BRCA* mutation type with OS and PFS was explored using a Cox regression model with *BRCA* mutation type as a covariate. Median PFS and OS were estimated by the Kaplan-Meier method. The HR and 95% CI were calculated separately for each cohort.

*BRCA* breast cancer susceptibility gene, *CI* confidence interval, *gBRCAm* germline *BRCA* mutation, *HER2-* human epidermal growth factor receptor 2-negative, *HR+* hormone receptor-positive, *HR* hazard ratio, *NE* not estimable, *OS* overall survival, *PFS* progression-free survival, *TNBC* triple-negative breast cancer
